# Amorphous Solid Dispersion Hydrogel Platform for Transdermal Delivery of Cannabidiol with Therapeutic Potential for Dermatitis

**DOI:** 10.3390/pharmaceutics18060666

**Published:** 2026-05-28

**Authors:** Badmaarag-Altai Chuluunbaatar, Yujin Jeong, Jieun Ok, Yujin Song, Jae Woon Son, Ji-Hyun Kang, Wonwoong Lee, Kyung Hyun Min

**Affiliations:** 1 School of Pharmacy, Jeonbuk National University, Jeonju 54896, Republic of Korea; 2 Department of Bionanotechnology and Bioconvergence Engineering, Graduate School, Jeonbuk National University, Jeonju 54896, Republic of Korea; 3 Institute of New Drug Development, Jeonbuk National University, Jeonju 54928, Republic of Korea; 4 Respiratory Drug Development Research Institute, Jeonbuk National University, Jeonju 54928, Republic of Korea; 5 College of Pharmacy, Woosuk University, Wanju 55338, Republic of Korea; 6 Research Institute of Pharmaceutical Sciences, Woosuk University, Wanju 55338, Republic of Korea

**Keywords:** amorphous solid dispersion, hydrogel, cannabidiol, skin disorders, anti-inflammatory

## Abstract

**Background/Objectives**: *Cannabis sativa* is the source of cannabidiol (CBD), a non-intoxicating phytocannabinoid with analgesic and anti-inflammatory qualities that has demonstrated therapeutic potential in inflammatory skin conditions like dermatitis. However, low bioavailability and poor water solubility restrict its topical application. This study attempted to improve CBD solubility and transdermal delivery using an amorphous solid dispersion (ASD)-based hydrogel system. **Methods**: CBD was stabilized in its amorphous form using an ASD strategy and incorporated into a hydrogel matrix. The CBD-ASD hydrogel was characterized by particle size analysis, scanning electron microscopy (SEM), Fourier-transform infrared spectroscopy (FT-IR), rheological assessment, swelling studies, and diffusion experiments using Franz cells. Biological evaluations included cytotoxicity testing in human dermal fibroblast (HDF) cells, wound-healing assays, RT-qPCR-based anti-inflammatory analysis, antioxidant activity (DPPH assay), and antibacterial testing against *Staphylococcus aureus*. **Results**: Physicochemical analyses confirmed successful amorphous dispersion of CBD within a stable hydrogel network. The formulation exhibited sustained drug release over 144 h, achieving 86.32% cumulative release with diffusion-controlled kinetics. Rheological and swelling properties demonstrated mechanical stability and hydration suitability for long-term topical application, while Franz diffusion studies confirmed effective transdermal permeation. The CBD-ASD hydrogel showed no cytotoxicity in HDF cells and significantly enhanced wound closure. It also downregulated pro-inflammatory cytokines including interleukin-6 (IL-6) and tumor necrosis factor-alpha (TNF-α). Additionally, the formulation demonstrated 65.63 ± 10.00% DPPH radical scavenging activity and over 99% antibacterial inhibition. **Conclusions**: The CBD-ASD hydrogel represents a stable, multifunctional delivery platform that overcomes CBD solubility limitations and enhances therapeutic efficacy for inflammatory skin diseases.

## 1. Introduction

The human body’s main defense mechanism is the skin, shielding against physical injury, microbial invasion, and chemical exposure [1]. Continuous exposure to environmental stressors renders the skin vulnerable to inflammatory dermatological disorders, including atopic dermatitis (AD), psoriasis, contact dermatitis, and acne, many of which are driven by dysregulated inflammatory responses [2,3]. These conditions are characterized by cytokine dysregulation, oxidative stress, and chronic inflammation, ultimately leading to impaired barrier function and recurrent clinical symptoms. Current therapeutic strategies largely depend on corticosteroids and systemic immunosuppressive agents. While these treatments effectively control inflammation, long-term use is frequently linked to both systemic and local side effects, including skin thinning, barrier impairment, and increased risk of infection [4,5]. This therapeutic limitation underscores the need for safer, localized anti-inflammatory interventions that minimize systemic toxicity.

The non-intoxicating phytocannabinoid cannabidiol (CBD), which comes from *Cannabis sativa*, is becoming more well-known for its analgesic, anti-inflammatory, and antioxidant qualities [6]. CBD modulates inflammatory signaling pathways and reduces pro-inflammatory cytokine expression, suggesting therapeutic relevance for AD and psoriasis [7]. Topical administration is particularly attractive for localized skin disorders; however, CBD’s low bioavailability, lipophilicity, and poor water solubility continue to limit its therapeutic application [8,9]. These physicochemical barriers restrict effective skin permeation and compromise therapeutic consistency, particularly in topical applications [10].

Transdermal delivery systems, particularly hydrogel-based platforms, have demonstrated considerable promise for localized and sustained drug release [11]. Hydrogels are networks of three-dimensional hydrophilic polymers that can hold significant volumes of water, thereby enhancing skin hydration and improving patient compliance [12,13]. Their tunable physicochemical properties allow precise modulation of drug release kinetics and skin permeation behavior, making them highly adaptable for dermatological applications [14]. Additionally, hydrogels provide a hydrated and structurally stable microenvironment that protects encapsulated drugs from premature degradation, thereby enhancing formulation stability and preserving bioactivity during transdermal administration [14,15]. Beyond hydration effects, hydrogels can reduce transepidermal water loss (TEWL) and establish an occlusive microenvironment that facilitates drug penetration [16,17]. Their rheological and adhesive characteristics can be engineered to prolong skin residence time, improving localized bioavailability [18]. Furthermore, advances in crosslinking strategies enable precise control over network density, mechanical integrity, and diffusion pathways, thereby optimizing therapeutic performance [19].

**Figure 1:**
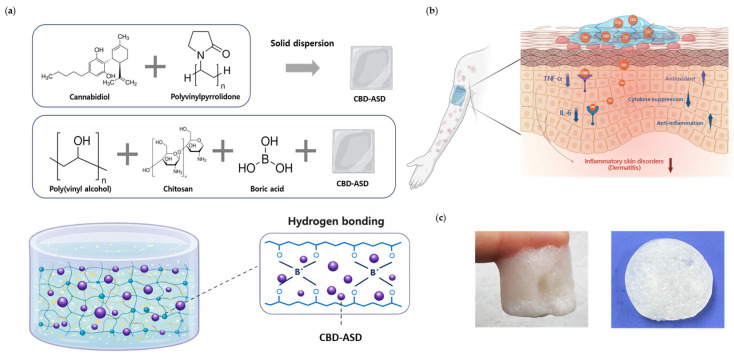
Schematic illustration of the fabrication process, network architecture, and proposed therapeutic mechanism of the CBD-ASD hydrogel. (**a**) Preparation of CBD-ASD using CBD and PVP, followed by incorporation into a PVA/chitosan/boric acid hydrogel matrix. The hydrogel network is stabilized by hydrogen bonding and borate–diol interactions. (**b**) Proposed topical delivery of CBD from the hydrogel and its anti-inflammatory effects in skin lesions. (**c**) Representative images of the actual prepared hydrogel.

Polyvinylpyrrolidone (PVP) is a hydrophilic and biocompatible polymer widely utilized in amorphous solid dispersion (ASD) systems due to its strong hydrogen-bonding capacity and ability to inhibit drug crystallization [20]. By stabilizing hydrophobic drugs in an amorphous form with high energy inside a polymeric matrix, ASD technology enhances apparent solubility, dissolution behavior, and thermodynamic activity while preventing recrystallization during storage and application [21,22,23,24,25]. Maintenance of supersaturation further promotes drug diffusion across biological membranes, which is particularly critical in transdermal systems where solubility directly governs permeation efficiency [25,26]. This approach is particularly advantageous for transdermal systems, where drug solubility critically influences skin permeation efficiency. Thus, converting CBD into its amorphous form and stabilizing it within a polymeric carrier represents a rational strategy to overcome its intrinsic solubility limitations (Figure 1).

While ASD–hydrogel systems have been reported in various pharmaceutical applications [27], research specifically focused on CBD delivery remains limited, particularly with respect to the simultaneous enhancement of dermal permeation and formulation stability. Despite the individual advantages of hydrogel-based systems and ASD technology, their combined application for CBD delivery has not been systematically investigated in the context of dermal permeation enhancement and formulation stability. In this study, PVA and chitosan were combined to construct a hydrogel matrix capable of supporting the incorporation of CBD-loaded ASD while maintaining hydrogel integrity and controlled drug release behavior. The combination of these polymeric components was selected based on their complementary physicochemical and mechanical properties reported in previous hydrogel delivery systems [28]. We hypothesized that incorporating ASD-stabilized CBD into a hydrogel matrix would synergistically improve drug solubility, physicochemical stability, and transdermal delivery efficiency. Accordingly, the purpose of this research was to create and evaluate a CBD-ASD hydrogel system as a formulation-based method to overcome the physicochemical barriers limiting topical CBD therapy. This integrated platform may provide a clinically translatable approach to alleviate dermatitis and skin inflammation.

## 2. Materials and Methods

### 2.1. Materials

Polyvinyl alcohol (PVA) (Product Number: 363146, Molecular Weight: 85,000–124,000 mol wt., Hydrolysis Rate: 99%), polyvinylpyrrolidone (PVP), Boric acid, Gallic acid (Product number: G7384), and Chitosan (Product number: 448869) were purchased from Sigma-Aldrich, St. Louis, MO, USA. CBD in solid form was obtained from the LED Agricultural Life Science Convergence Technology Research Center of Jeonbuk National University (Jeonju, Republic of Korea). Acetonitrile (Product number: UN1648) was obtained from J.T.Baker, Seoul, Republic of Korea. The supplier of formic acid (Product number: 64186) was Thermochemical in Berlin, Germany. The Milli-Q^®^ of Burlington, MA, USA was the source of the distilled water used in the entire investigation.

### 2.2. Methods

#### 2.2.1. ASD Film Preparation

Amorphous solid dispersion (ASD) films were prepared using polyvinylpyrrolidone (PVP) with or without CBD. For the CBD-loaded ASD film (CBD-ASD), 750 mg of PVP and the desired amount of CBD were dissolved in ethanol under continuous stirring until the solution was homogeneous. The outcome of the solution was evenly spread onto a pre-cleaned silicon mold to form a uniform film. The solvent was evaporated at 40 °C for 24 h under controlled conditions to get the ASD film. The films were dried and then carefully taken out of the mold and stored in a desiccator to prevent moisture absorption and potential recrystallization. The prepared films were subsequently used for further characterization.

#### 2.2.2. Formulation of Hydrogels

Polyvinyl alcohol (PVA) was dissolved in distilled water to create a 15% (*w*/*v*) solution. After stirring the mixture at 400 rpm, it was heated to 90 °C for an hour, until it became completely clear, indicating complete dissolution of the polymer. The solution was allowed to cool to room temperature under ambient conditions. Separately, the prepared ASD films with or without CBD were mixed with 5 mL of the cooled PVA solution. To this mixture, 53 mg of boric acid and 20 mg of chitosan were added as cross-linking and stabilizing agents, respectively. Chitosan acts as a stabilizer by forming a robust network of intermolecular hydrogen bonds with the PVA chains. This interaction enhances the structural integrity and regulates the swelling behavior of the composite matrix, thereby addressing the mechanical instability and high solubility associated with pure PVA hydrogels [29,30]. The components were thoroughly mixed to obtain a homogeneous dispersion. The formulation compositions are presented in Table 1. The final mixture was transferred into a previously cleaned weighing plate and allowed to gel under regulated circumstances, thereby forming the hydrogel matrix.

#### 2.2.3. Morphological Characterization by FE-SEM

A field emission scanning electron microscope (FE-SEM, GeminiSEM 300, Carl Zeiss, Oberkochen, Germany) was employed to observe the surface morphology. Conductivity was ensured by sputter-coating the samples with a fine layer of platinum before imaging. The coating process was carried out under vacuum conditions to ensure uniform metal deposition. FE-SEM imagery was performed at 3 kV at various magnifications to characterize the surface appearance and structure.

#### 2.2.4. Crystallinity Analysis by PXRD

The materials’ crystalline properties were examined using powder X-ray diffraction (PXRD) analysis. Diffraction patterns were captured using a D8 ADVANCE X-ray diffractometer (Bruker, AXS, Karlsruhe, Germany). The samples were directly mounted on the sample holder without additional processing. PXRD experiments were performed at 40 kV and 40 mA using Cu Kα radiation. Data collection spanned a 2θ range of 0–90° with a 0.04° step size and 0.5 s integration time per step.

#### 2.2.5. Thermal Characterization by DSC

The thermal characteristics of the materials were examined using differential scanning calorimetry (DSC). Before analysis, hydrogel-type samples were freeze-dried to remove residual moisture. Samples were packed in an aluminum pan with around 15 mg and analyzed using a DSC 2500 instrument by TA Instruments in New Castle (DE, USA). Heat flow was measured as a function of temperature while the samples underwent heating at a steady rate of 10 °C per minute from 0 to 180 °C.

#### 2.2.6. FT-IR Analysis

Using a Spectrum 3 FT-IR spectrometer (PerkinElmer, Shelton, CT, USA) fitted with an attenuated total reflectance (ATR) accessory for zinc selenide (ZnSe), the chemical structure of the hydrogels was examined. The ATR-FTIR spectra were acquired by averaging 64 scans across a wavenumber range of 4000–500 cm^−1^ at a resolution of 4 cm^−1^. The acquired spectra were utilized to determine distinctive functional groupings and possible interactions among the polymer components and cross-linking agents within the hydrogel matrix.

#### 2.2.7. Rheology Behavior

The rheological characterization of the hydrogels was performed using an Anton Paar MCR 92 rheometer (Graz, Austria). Measurement parameters were optimized using a 40 mm diameter cone-and-plate geometry with a 1° cone angle and a truncated gap of 0.1 mm. The viscoelasticity of the hydrogels was assessed at 25 °C via amplitude sweep tests (0.1–200% strain). By recording the storage (G′) and loss (G″) moduli as functions of strain, the linear viscoelastic limit was determined, specifically identifying the G′ = G″ crossover point as the threshold of network deformation.

#### 2.2.8. Swelling Behavior

The hydrogels’ swelling ratio was used to evaluate their swelling behavior. First, the hydrogels were dried to a constant weight in a desiccator, and an analytical balance was used to accurately record the dry weight (W_d_). The formulation of hydrogels was achieved through incubation in PBS (pH 7.4) at 37 °C for physiological condition simulation. To ensure measurement precision, samples were removed from the PBS at 1, 3, 6, 9, 12, and 24 h, followed by the removal of surface water via gentle blotting with filter paper. The swollen weight (W_t_) of each hydrogel sample was then recorded.

The following formula was used to get the swelling ratio:

(1) $$\mathrm{Swelling}\;\mathrm{ratio}\;(\%)=\left[\frac{\left(W_{t}-W_{d}\right)}{W_{d}}\right]\times 100$$

where W_d_ is the hydrogel’s dry weight, and W_t_ is its swelled weight.

#### 2.2.9. In Vitro Release Kinetics

CBD release from the CBD-loaded ASD hydrogel in vitro was evaluated using a dynamic dissolution method. The hydrogel sample was placed in pH 7.4 PBS containing 1% Tween 80 and shaken continuously at 150 rpm while being incubated at 37 °C. To maintain constant sink conditions, 40 mL of the release medium was periodically removed and replaced with fresh PBS containing 1% Tween 80 at intervals ranging from 1 to 144 h. After combining 0.7 mL of the obtained sample with 0.7 mL of acetonitrile (ACN), the mixture was centrifuged for 10 min at 10,000 rpm. High-performance liquid chromatography (HPLC) was used to collect and analyze the supernatant to estimate the CBD concentration. The cumulative drug release was then represented as a percentage over time. To investigate the drug release mechanism, the cumulative release data were further fitted to five mathematical kinetic models: Zero-order (Equation (2)), First-order (Equation (3)), Higuchi (Equation (4)), Hixson–Crowell (Equation (5)), and Korsmeyer–Peppas (Equation (6)). The coefficient of determination (*R*^2^) was employed to evaluate the goodness of fit for each model.

(2) $$Q_{t}=k_{0}t$$

(3) $$log\;Q_{t}=log\;Q_{0}-(k_{1}t/2.303)$$

(4) $$Q_{t}=k_{H}\;t^{1/2}$$

(5) $${Q_{0}}^{1/3}-{Q_{t}}^{1/3}=k_{HC}\;t$$

(6) $$M_{t}/M_{\infty }=k_{KP}\;t^{n}$$

#### 2.2.10. In Vitro Permeation

Transmembrane diffusion of CBD from the CBD-ASD hydrogel was evaluated using Franz diffusion cells (Lab FINE, Gunpo-si, Republic of Korea). A Strat-M^®^ synthetic membrane was mounted with a 1.77 cm^2^ effective diffusion space between the donor and receptor chambers. To maintain sink conditions, 12 mL of pH 7.4 PBS containing 1% Tween 80 was added to the receptor compartment. The receptor media was continuously swirled at 600 rpm and kept at 37 ± 0.5 °C. The hydrogel formulation was added to the donor chamber in an amount of around 1 g. One milliliter aliquots were removed from the receptor compartment at predefined intervals (1, 2, 6, 12, 24, 48, and 72 h) and replaced with an equivalent volume of new receptor media. HPLC analysis was employed to quantify the samples. The steady-state flux (Jss) was subsequently determined from the slope of the cumulative permeation curve’s linear region.

#### 2.2.11. Animals

All animal experiments were conducted in strict accordance with the relevant guidelines and the Principles of Laboratory Animal Care, with approval from the Institutional Animal Care and Use Committee (IACUC) of Jeonbuk National University, Republic of Korea. Eight-week-old male Sprague–Dawley (SD) rats (Samtako, Gyeonggi-do, Republic of Korea) were utilized for the study. The animals were housed under controlled environmental conditions (12 h light/dark cycle), with unrestricted access to food and water.

#### 2.2.12. Ex Vivo Skin Retention Study

Full-thickness dorsal skin from SD rats was used for the permeation investigations. An electric clipper was used to delicately remove the dorsal hair, and the removed skin was gathered. The skin’s remaining connective tissues and subcutaneous fat were carefully removed. Before being used, the processed skin samples were kept at −20 °C. Prior to the experiment, the frozen skin was thawed for 24 h at 4 °C and equilibrated for 3 h at 37 °C in 0.1 M PBS (pH 7.4). The Franz diffusion cell’s donor and receptor compartments were separated by the moist skin, with the stratum corneum (SC) towards the donor chamber. To preserve sink conditions, 0.1 M PBS, a pH 7.4, with 1% Tween 80 was added to the receptor chamber (12 mL). The temperature of the receptor media was kept at 37 °C using a circulating water bath. The tape-stripping method was used to assess drug deposition inside the dermis following the 72-h permeation investigation. Adhesive tape (3M) was used to gradually remove the SC. The initial three strips were thrown away to remove residual formulation on the skin surface, and strips 4–20 were retained for analysis. All SC tape strips and the remaining dermal tissue were then cut into small pieces, immersed in the HPLC mobile phase, and extracted by sonicating for 30 min the deposited drug before quantitative analysis.

#### 2.2.13. Antioxidant Activity

A DPPH radical scavenging test was used to assess the CBD-loaded ASD hydrogel’s antioxidant qualities. A solution of 0.1 mM DPPH was made in 100% ethanol. For the experiment, 50 μL of DPPH solution was combined with 100 μL of drug release samples collected from the hydrogels at different time points. The mixtures were incubated at room temperature in the dark for half an hour. After incubation, the absorbance of the samples (A_sample_) was assessed at 517 nm by a microplate reader. A control solution without hydrogel was used to determine the baseline absorbance (A_control_).

The percentage of DPPH radical scavenging activity was calculated by the equation:

(7) $$\mathrm{Radical}\;\mathrm{scavenging}\;\mathrm{activity}\;(\%)=\left[\frac{\left(A_{control}-A_{sample}\right)}{A_{control}}\right]\times 100$$

#### 2.2.14. Antibacterial Activity

The colony-forming unit (CFU) counting method was used to calculate the amount of viable bacterial cells. At each dilution phase, 100 μL of Staphylococcus aureus (ATCC 29213), a Gram-positive bacterial suspension (1 × 10^6^ CFU/mL), was transferred into 900 μL of sterile broth to create a serial dilution (1:10). Dilutions ranging in the range of 10^−6^ to 10^−9^ were prepared to obtain countable colonies. From each dilution, the bacterial suspension (100 μL) was applied to nutrient agar plates using a sterile spreader. The plates were incubated overnight at 37 °C. Following incubation, visible colonies were enumerated on plates exhibiting between 30 and 300 CFUs.

The bacterial viability was quantified in CFU/mL by the following equation:

(8) $$\mathrm{CFU}/\mathrm{mL}=\frac{Number\;of\;colonies}{Total\;Dilution\;Factor}$$

The antibacterial ratio was derived using the equation below:

(9) $$\mathrm{Antibacterial}\;\mathrm{ratio}\;(\%)=\left[\frac{\left(C_{control}-C_{sample}\right)}{C_{control}}\right]\times 100$$

where C_control_ and C_sample_ represent the colony counts of the control and treated samples, respectively.

#### 2.2.15. Cell Culture and Conditioned Medium

The primary adult human dermal fibroblasts (HDF) used in this study were purchased from the American Type Culture Collection (ATCC; PCS-201-012, Manassas, VA, USA) and cells were cultured in glucose-free Dulbecco’s Modified Eagle Medium (DMEM) lacking sodium pyruvate with containing 10% fetal bovine serum (FBS) and antibiotics (penicillin 100 U/mL and streptomycin 100 µg/mL) at 37 °C in a humidified 5% CO_2_ environment.

For the preparation of hydrogel-conditioned media, accurately weighed CBD-ASD hydrogels were immersed in complete culture medium, then incubated for 24 h at 37 °C with mild agitation (100 rpm). The obtained conditioned media corresponded to final CBD concentrations of 5 or 10 µM. As a formulation control, ASD hydrogels without CBD were processed in the same manner and at an equivalent mass to obtain a control conditioned medium.

#### 2.2.16. Cytotoxicity Assay

The biological safety of the CBD-ASD hydrogel systems was evaluated through indirect contact assays using extract-enriched media. HDF cells were seeded into 96-well microplates at a seeding concentration of 5 × 10^3^ cells/well and maintained overnight to facilitate cell attachment under optimal culture conditions. Following the initial period, the incubation medium was exchanged for conditioned media prepared from CBD-ASD hydrogels, and the cultures were incubated for an additional 24 h. Cell viability was assessed using a Cell Counting Kit-8 (CCK-8; Dojindo Molecular Technologies, Kumamoto, Japan) in accordance with the manufacturer’s instructions. Post-incubation, 10 µL of CCK-8 solution was introduced to each well, followed by a further incubation at 37 °C for 1–4 h. Finally, the optical density was determined at 450 nm via a microplate spectrophotometer.

#### 2.2.17. Wound Healing Assay

To assess cellular motility, HDF cells were seeded into 6-well culture dishes at a density of 1 × 10^5^ cells/well and cultivated until reaching sub-confluence. A consistent linear wound was introduced into the integrated monolayer using a sterile 200 µL pipette tip, establishing a denuded area. Detached cells and debris were meticulously eliminated through double washing with PBS (pH 7.4). Except for the negative control, an inflammatory state was triggered by treating the cells with 2 µg/mL lipopolysaccharide (LPS) for a 2 h duration. After this induction, the cells were exposed to either CBD-ASD hydrogel-conditioned media (5 or 10 µM) or ASD hydrogel control media for a further 18 h. Migration and wound closure were assessed by capturing images at predetermined time points using a light microscope at 4× magnification, and the relative closure area was analyzed accordingly.

#### 2.2.18. RT-qPCR Analysis

HDF cells were cultured in 6-well microplates and subjected to lipopolysaccharide (LPS, 2 µg/mL) exposure for 2 h to trigger an inflammatory cascade. Post-induction, the cells were incubated with extract media from CBD-ASD hydrogels (5 or 10 µM) or ASD hydrogel controls for a further 18 h. Extraction of total RNA from both experimental and reference groups was performed using TRIzol reagent (Thermo Fisher Scientific, Waltham, MA, USA) following the manufacturer’s guidelines. The yield and integrity of the isolated RNA were quantified via ultramicro-spectrophotometry (NanoDrop system, Thermo Fisher Scientific). Thereafter, 1 µg of total RNA was converted into complementary DNA (cDNA) through reverse transcription using a high-capacity cDNA synthesis kit. Real-time fluorescence-based quantitative PCR (RT-qPCR) was executed with SYBR Green master mix (Enzynomics, Daejeon, Republic of Korea) on a QuantStudio real-time PCR platform (Thermo Fisher Scientific). Target gene transcripts for pro-inflammatory cytokines were normalized against the housekeeping gene GAPDH. The relative fold-change in gene expression was determined via the comparative 2^−ΔΔCt^ method [31]. The specific oligonucleotide sequences for the primers utilized are listed in Table 2.

#### 2.2.19. Statistical Analysis

Unless otherwise noted, all experimental procedures were executed in triplicate to ensure reproducibility. Data are reported as the mean ± standard deviation (SD). Quantitative comparisons and statistical significance were determined using GraphPad Prism (v. 8.0; GraphPad Software, San Diego, CA, USA). For multi-group comparisons, a one-way analysis of variance (ANOVA) was employed, complemented by Tukey’s post hoc test for inter-group differences. Statistical relevance was defined at a threshold of *p* < 0.05.

## 3. Results and Discussion

### 3.1. Physicochemical Characterization of CBD Amorphous Solid Dispersions

**Figure 2:**
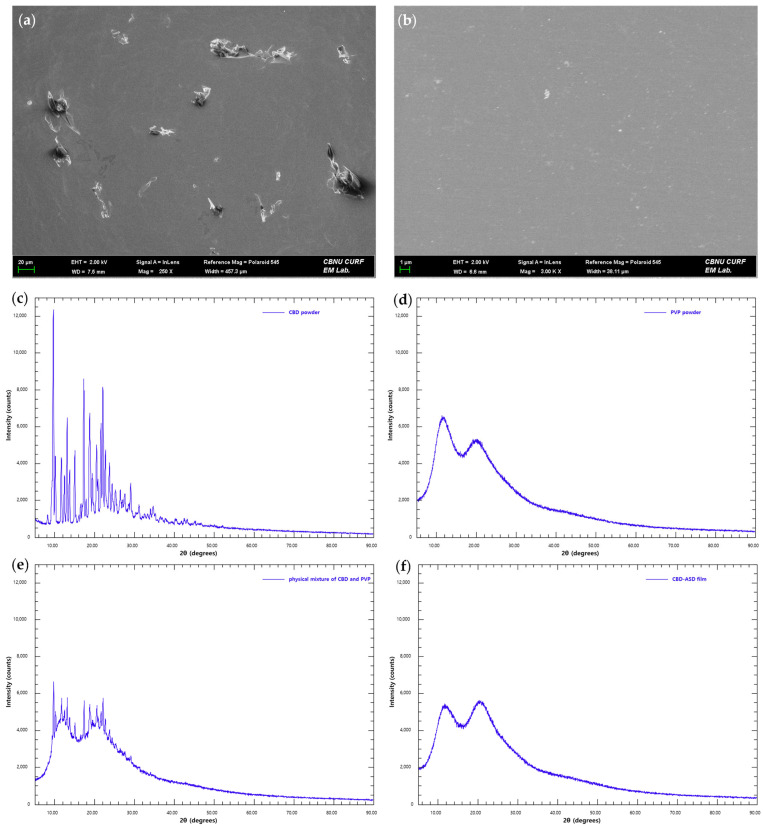
Surface morphology of (**a**) CBD powder and (**b**) CBD-ASD by SEM. PXRD patterns of (**c**) CBD powder, (**d**) PVP powder, (**e**) the physical mixture of CBD and PVP, and (**f**) CBD-ASD film, demonstrating the reduced crystallinity of CBD after amorphous solid dispersion formation.

The surface morphology of crystalline CBD and its amorphous solid dispersion was evaluated using FE-SEM. Pure CBD exhibited well-defined crystalline particles with sharp edges and angular geometries, consistent with its ordered lattice structure (Figure 2a). In contrast, CBD-ASD displayed a smooth and homogeneous surface without visible crystalline features (Figure 2b), indicating disruption of the original crystal architecture following incorporation into the polymer matrix. To further elucidate solid-state transformation, PXRD analysis was performed (Figure 2c–f). Pure CBD showed distinct and intense diffraction peaks characteristic of its crystalline nature; in contrast, PVP displayed an amorphous material-typical broad halo pattern. Importantly, the diffraction profile of CBD-ASD lacked the characteristic crystalline peaks observed in pure CBD, confirming successful conversion to an amorphous form within the polymer matrix. In contrast, the physical mixture of CBD and PVP retained residual crystalline reflections, suggesting that simple blending is insufficient to achieve complete amorphization. These findings collectively demonstrate that the solid dispersion process enabled effective molecular dispersion of CBD.

The absence of crystalline reflections in CBD-ASD further suggests the presence of intermolecular interactions between CBD and PVP, which likely suppress molecular rearrangement and inhibit recrystallization. Transition to the amorphous state is expected to enhance dissolution performance by eliminating the energetic barrier associated with crystal lattice disruption. This interpretation aligns with recent studies reporting polymer-mediated stabilization of amorphous dispersions through restricted molecular mobility and enhanced thermodynamic activity [32,33]. Together, these results substantiate the successful amorphization of CBD and support its potential for improved solubility-driven performance in subsequent formulations.

### 3.2. Morphological Characterization

**Figure 3:**
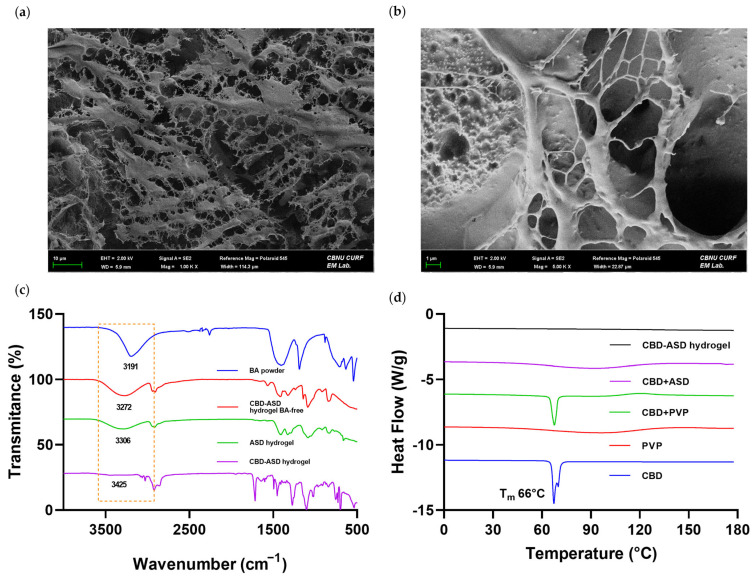
Morphological and physicochemical characterization of the hydrogel formulations. SEM image of the hydrogel at (**a**) ×1000 and (**b**) ×5000 magnification. (**c**) FT-IR spectra of boric acid powder, CBD-ASD hydrogel without boric acid, ASD hydrogel, and CBD-ASD hydrogel. (**d**) DSC thermograms of CBD, PVP, a physical mixture of CBD and PVP, CBD-ASD film, and CBD-ASD hydrogel.

Surface morphology of lyophilized CBD-ASD hydrogels was examined by FE-SEM, and representative micrographs are presented in Figure 3a,b. The hydrogels exhibited a coarse, extensively porous architecture with interconnected pore structures, indicative of a well-developed crosslinked polymer network. The uniform distribution of pores suggests structural homogeneity throughout the matrix. Porous microstructures are widely recognized as critical determinants of hydrogel performance, as pore size and network density directly influence water uptake, swelling behavior, and mass transport properties [34]. The interconnected architecture observed in the present system is expected to facilitate the diffusion of encapsulated drug molecules while maintaining structural integrity. Similar porous hydrogel networks have been reported to promote sustained release profiles by enabling controlled solvent penetration and drug diffusion through the polymeric matrix [35]. The FE-SEM findings confirm the successful formation of a structurally organized hydrogel network suitable for supporting controlled release of CBD-ASD within a dermal delivery platform.

### 3.3. FT-IR Spectroscopic Analysis

The chemical interactions within the hydrogel formulations were investigated by FT-IR spectroscopy (Figure 3c). In all hydrogel formulations, an extensive absorption envelope was observed within the spectral range of 3200–3600 cm^−1^, which is attributable to the stretching modes of intermolecularly hydrogen-bonded hydroxyl (–OH) groups in the PVA backbone. These characteristic bands confirm preservation of the PVA network structure after ASD incorporation. The BA powder displayed distinct borate-related absorption bands in the lower wavenumber region. Upon incorporation of boric acid into the CBD-ASD hydrogel, noticeable attenuation of the –OH stretching band was observed, indicating involvement of hydroxyl groups in intermolecular interactions. The –OH stretching band shifted progressively from 3272 cm^−1^ (BA-free ASD hydrogel) to 3306 cm^−1^ (ASD hydrogel, a shift of 34 cm^−1^) and 3425 cm^−1^ (CBD-ASD hydrogel, a shift of 153 cm^−1^), relative to the BA powder reference at 3191 cm^−1^. This progressive shift indicates increasing involvement of hydroxyl groups in borate–diol interactions with growing formulation complexity; the larger shift observed in the CBD-ASD hydrogel likely reflects additional hydrophobic interactions introduced by CBD. In addition, subtle spectral shifts in the borate-related region (~600–700 cm^−1^) suggest the formation of borate–diol complexes between boric acid and the hydroxyl groups of PVA, confirming successful dynamic crosslinking within the hydrogel matrix. In contrast, the BA-free hydrogels (ASD hydrogel and CBD-ASD hydrogel BA-free) did not exhibit these borate-associated spectral features, supporting that the observed structural changes originate from borate-mediated crosslinking rather than drug incorporation. Similar borate-mediated dynamic covalent interactions in PVA hydrogels have been reported to regulate network stability and swelling behavior [36,37].

### 3.4. DSC Analysis

As shown in Figure 3d, the thermal characteristics were investigated using DSC. The thermographic analysis of pristine CBD revealed a characteristic melting endotherm near 66 °C, consistent with its highly ordered crystalline structure. This sharp endotherm indicates a high degree of crystallinity with a defined melting point. Conversely, the complete disappearance of this endothermic transition in the CBD-ASD system suggests the loss of long-range crystalline order. This indicates that CBD was successfully transformed into an amorphous state and homogeneously distributed within the polymeric environment. The resulting thermogram for the ASD showed a smooth baseline without any detectable phase transitions within the analyzed temperature range. Similarly, the CBD-ASD hydrogel samples did not exhibit a distinct melting peak corresponding to CBD, suggesting that the drug remained in a non-crystalline state after incorporation into the hydrogel network. The broad endothermic features observed at higher temperatures are attributed to the evaporation of residual moisture and the thermal relaxation of the hydrogel network, which are typical for such polymeric systems, rather than any drug-related melting events. The disappearance of the characteristic CBD melting peak implies successful amorphization of the drug within the polymeric system, which may enhance its apparent solubility and dissolution behavior. These results support the PXRD analysis; the absence of distinct crystalline peaks confirms the successful formation of an amorphous solid dispersion of CBD.

### 3.5. Rheological Behavior of Hydrogels

**Figure 4:**
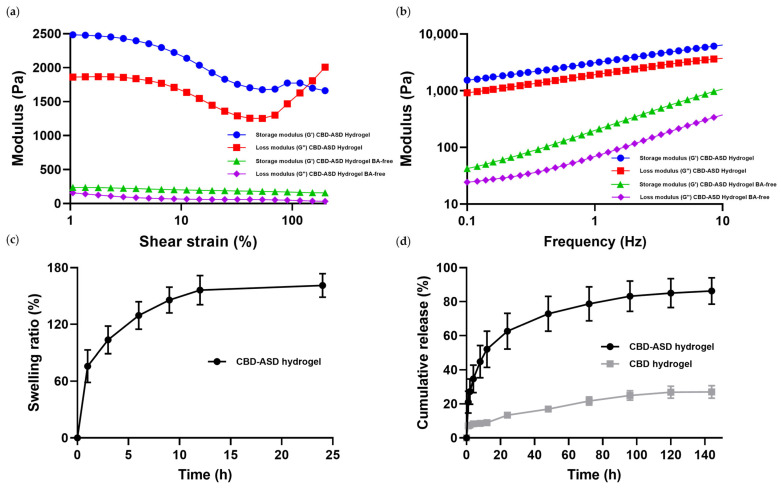
Rheological and mechanical characterization of the hydrogels. (**a**,**b**) Strain and frequency sweep measurements of CBD-ASD hydrogel and BA-free CBD-ASD hydrogel, displaying the loss (G″) and storage (G′) moduli. (**c**) The CBD-ASD hydrogel’s swelling ratio. (**d**) CBD and CBD-ASD hydrogel in vitro release profiling. Data are reported as mean ± SD (*n* = 3).

Since the rheological behavior of standalone PVA- and chitosan-based hydrogels has been extensively described in the literature, this study primarily evaluated the viscoelastic performance of the integrated hybrid matrix in relation to ASD-stabilized CBD incorporation and delivery. Dynamic rheological analysis was employed to assess the viscoelastic signatures of the hydrogel samples, specifically focusing on the frequency dependence of the elastic modulus, and the results are presented in Figure 4a,b. The amplitude sweep test (0.1–400% strain) demonstrated that the storage modulus (G′) was consistently superior to the loss modulus (G″), evidencing the establishment of a well-defined three-dimensional hydrogel network. Notably, the crossover point (G′ = G″) of the CBD-ASD hydrogel occurred at a strain beyond 100%, indicating enhanced deformation resistance compared with the BA-free CBD-ASD hydrogel. This delayed structural yielding suggests a higher crosslinking density mediated by borate–diol interactions, which reinforce the hydrogel network. In the frequency sweep test (0.01–10 Hz, 1% strain), G′ consistently exceeded G″ across the entire frequency range, with minimal frequency dependence, indicating the dominance of elastic behavior and the presence of a well-established crosslinked network structure. This behavior is characteristic of well-established crosslinked systems and indicates solid-like dominance under dynamic mechanical conditions. Similar rheological profiles have been associated with improved mechanical stability and controlled drug diffusion in transdermal hydrogel platforms [38]. The enhanced elastic dominance and delayed crossover point indicate that boric acid incorporation reinforces network integrity and mechanical stability, supporting sustained dermal drug delivery.

### 3.6. Swelling Behavior of CBD-ASD Hydrogel

The CBD-ASD hydrogel’s swelling profile was assessed in PBS at a pH of 7.4. (Figure 4c). A time-dependent increase in swelling ratio was observed, with a marked rise within the first 3–6 h (reaching approximately 130%), followed by a more gradual increase until 24 h (~160%), indicating attainment of near-equilibrium hydration. Swelling behavior in crosslinked polymeric hydrogels is driven by initial solvent diffusion into the network, followed by polymer chain relaxation constrained by crosslink density [39]. The initial rapid expansion reflects efficient water ingress into the interconnected porous network, whereas the slower swelling phase at later time points suggests progressive relaxation of constrained polymer chains.

This controlled expansion and the resulting dimensional stability are primarily governed by the synergistic interaction between the PVA and chitosan chains. While pure PVA networks are known for their high solubility and tendency toward excessive swelling or structural loss in aqueous media [40], the introduction of chitosan effectively stabilizes the matrix via extensive intermolecular hydrogen bonding [41]. This structural reinforcement provides the necessary integrity for sustained drug release, overcoming the inherent limitations typically associated with single-polymer PVA systems. Controlled swelling without excessive expansion has been linked to improved dimensional stability and sustained drug-release performance in hydrogel drug-delivery systems [42].

### 3.7. Drug Release and Transdermal Permeation of CBD-ASD Hydrogel

The extreme hydrophobicity of CBD poses a significant challenge for its direct incorporation into aqueous single-polymer hydrogel matrices [8,43]. Without stabilization by an ASD system, crystalline CBD tends to aggregate and distribute non-uniformly within the hydrogel matrix, potentially resulting in poor drug loading and inconsistent release behavior [44]. The CBD release profiles from the hydrogels in vitro demonstrated markedly different behaviors between the pure CBD and ASD formulations (Figure 4d). The pure CBD-loaded hydrogel exhibited limited release (~8% over 144 h), consistent with the low aqueous solubility and hindered diffusivity of crystalline CBD. In contrast, the CBD-ASD hydrogel exhibited rapid initial release (~62% within 24 h), followed by prolonged, sustained release up to ~86% at 144 h, reflecting enhanced dissolution and diffusivity associated with amorphization of the drug in the ASD [45]. To further investigate the release mechanism, cumulative release data from both formulations were fitted to multiple kinetic models, including zero-order, first-order, Higuchi, Hixson–Crowell, and Korsmeyer–Peppas models (Table 3). Among the tested models, the Korsmeyer–Peppas model showed the best fit for the CBD-ASD hydrogel (*R*^2^ = 0.980), while the Higuchi model provided a better fit for the pure CBD hydrogel (*R*^2^ = 0.974). The higher *R*^2^ value observed for the CBD-ASD hydrogel indicates more consistent, controlled release behavior after ASD incorporation.

**Figure 5:**
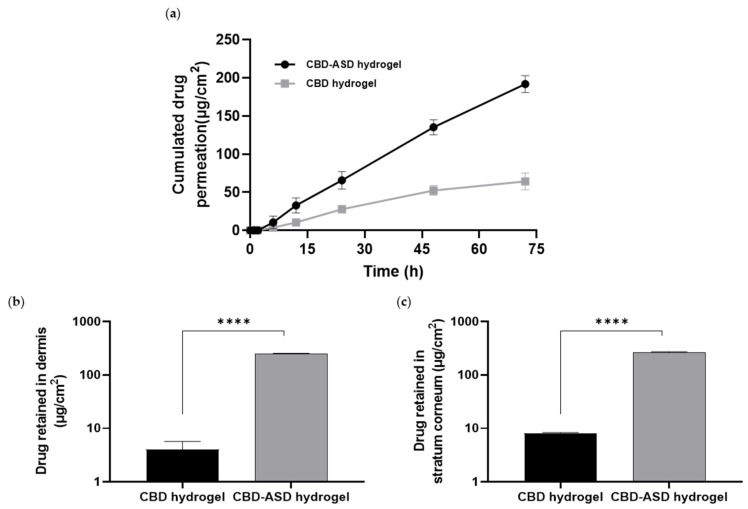
In vitro permeation and ex vivo skin retention of CBD hydrogel and CBD-ASD hydrogel. (**a**) Time-dependent permeation profiles of the hydrogels across a membrane. Amount of CBD retained in (**b**) the dermis and (**c**) the stratum corneum of rat skin following 72 h of Franz diffusion experiments. The data are all displayed as mean ± SD (*n* = 3). **** *p* < 0.0001 indicates statistical significance when compared to the CBD hydrogel group.

In vitro permeation studies using Strat-M membranes in Franz diffusion cells demonstrated the enhanced transmembrane delivery of CBD from the ASD-based hydrogel formulation. After 72 h, the cumulative permeation of CBD from the pure CBD hydrogel was 64.28 ± 11.14 µg/cm^2^, whereas the ASD-loaded hydrogel significantly increased permeation to 195.55 ± 3.74 µg/cm^2^ (Figure 5a). Enhanced permeation is attributed to the elevated thermodynamic activity of the amorphous drug and to improved availability for diffusion across the membrane, both known drivers of passive transdermal transport [46].

To further evaluate dermal delivery behavior, ex vivo permeation experiments were performed using rat dorsal skin (Figure 5b,c). Following the 72-h diffusion period, substantial amounts of CBD were retained within the skin layers when delivered from the CBD-ASD hydrogel. The retained drug levels were 267.45 ± 4.86 µg/cm^2^ in the stratum corneum (SC) and 249.27 ± 5.35 µg/cm^2^ in the dermis, indicating effective partitioning of CBD into both the outer barrier layer and deeper dermal tissue. The enhanced skin deposition observed with the ASD hydrogel suggests improved drug solubilization and thermodynamic driving force for skin uptake. This enhanced deposition and permeation can be attributed to two synergistic mechanisms. First, the amorphous state of CBD within the ASD matrix eliminates crystalline lattice energy, thereby increasing the thermodynamic activity and chemical potential of the drug, which serves as the primary driving force for diffusion across the stratum corneum. Second, the hydrogel matrix provides sustained hydration to the skin surface, which is known to swell and disorganize the lipid bilayers of the stratum corneum, reducing its barrier resistance and increasing the effective diffusion coefficient of lipophilic compounds such as CBD. Collectively, these findings indicate that ASD-mediated amorphization significantly enhances both transmembrane permeation (in vitro) and dermal drug retention (ex vivo), supporting the potential of the CBD-ASD hydrogel as an effective transdermal delivery platform.

### 3.8. Antioxidant Potential of CBD-ASD Hydrogel

**Figure 6:**
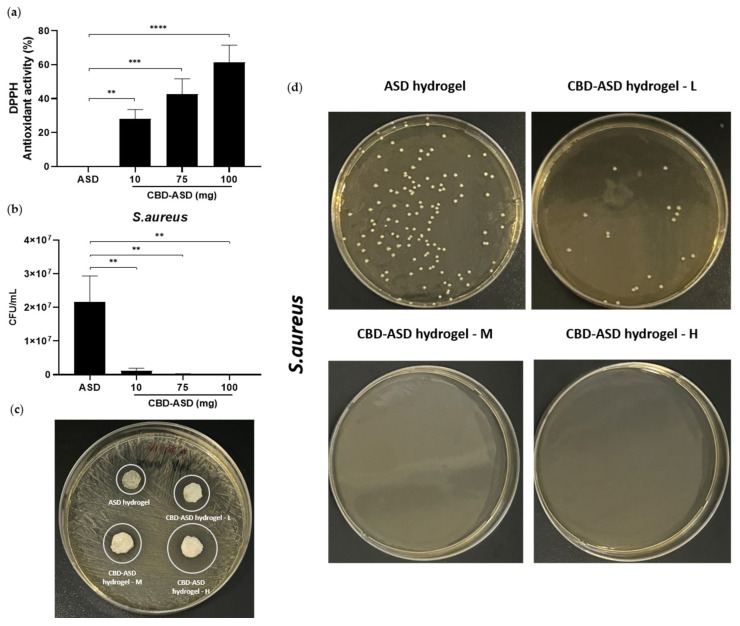
Antioxidant and antibacterial activities of the hydrogel formulations against *S. aureus*. (**a**) DPPH radical scavenging activity of ASD hydrogel and CBD-ASD hydrogels at different concentrations. (**b**) Quantitative antibacterial activity against *S. aureus*. (**c**) Representative inhibition zones produced by each formulation. (**d**) Representative agar plate images after 24 h of incubation. For all assays, CBD-ASD hydrogel formulations at low (L, 10 mg), medium (M, 75 mg), and high (H, 100 mg) concentrations were compared with ASD hydrogel as the control. All data are presented as mean ± SD (*n* = 3). Statistical significance is indicated as ** *p* < 0.01, *** *p* < 0.001, and **** *p* < 0.0001 compared to the ASD hydrogel.

The DPPH radical scavenging experiment was used to evaluate the hydrogels’ antioxidant capacity (Figure 6a). The findings showed that when CBD-ASD loading increased, the antioxidant effect increased in a concentration-dependent manner. Hydrogels containing 10 mg, 75 mg, and 100 mg of CBD-ASD exhibited radical scavenging activities of 31.25 ± 5.41%, 37.50 ± 9.02%, and 65.63 ± 10.00%, respectively. In contrast, the blank ASD hydrogel showed negligible scavenging activity, confirming that the antioxidant effect originates from the incorporated CBD. The enhanced scavenging activity is attributed to the phenolic hydroxyl groups of CBD, which donate hydrogen atoms to stabilize DPPH radicals and terminate free radical chain reactions. The observed dose-dependent response aligns with previous reports demonstrating that CBD exhibits significant free radical scavenging capacity due to its phenolic structure and electron-donating properties [47,48]. The incorporation of CBD-ASD into the matrix of hydrogel, therefore, not only enhances drug dispersion but also confers intrinsic antioxidant functionality. Such antioxidant activity is particularly advantageous in dermal applications, where oxidative stress contributes to inflammation, delayed wound healing, and skin barrier disruption.

### 3.9. Antibacterial Activity of CBD-ASD Hydrogel

The bactericidal performance of the CBD-ASD hydrogels was characterized against *Staphylococcus aureus* utilizing the standard agar plate colony-counting technique, with the results illustrated in Figure 6b–d. The formulations were prepared with increasing CBD-ASD loadings: L (10 mg), M (75 mg), and H (100 mg). All CBD-ASD hydrogels showed a pronounced reduction in viable bacterial colonies relative to the untreated control, with antibacterial rates exceeding 99%. In contrast, the ASD hydrogel exhibited negligible antibacterial activity. A concentration-dependent enhancement in antibacterial performance was observed. Consistent with these findings, the agar diffusion assay showed increasing inhibition zone diameters with higher CBD-ASD loading (Table 4). The H formulation (100 mg) exhibited the most pronounced bactericidal effect. The strong activity of CBD against *S. aureus* has been attributed to disruption of cytoplasmic membrane integrity and interference with essential macromolecular synthesis pathways [49,50]. These findings suggest that adding CBD-ASD into the hydrogel matrix confers potent antibacterial functionality, with higher drug loading further enhancing bactericidal efficacy.

### 3.10. Cell Viability of CBD-ASD Hydrogel

**Figure 7:**
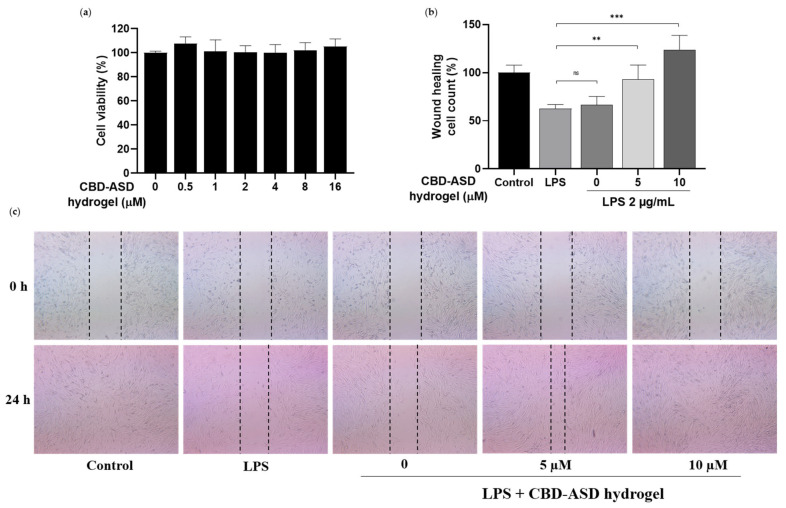
CBD-ASD hydrogels’ effects on HDF cell cytotoxicity and wound healing. (**a**) HDF cell viability following incubation with various concentrations of CBD-ASD hydrogel conditioned medium. (**b**) Quantitative analysis of wound healing in HDF cells stimulated by LPS. For the wound-healing assay, except for the negative control group, cells were initially exposed to LPS (2 µg/mL) for 2 h and then cultured for an additional 18 h in CBD-ASD hydrogel-conditioned medium. (**c**) Representative images showing cell migration and closure of the wounded area (4× magnification). All microscopic images were obtained at the same magnification. All data are presented as mean ± SD (*n* = 3). Statistical significance is indicated as ** *p* < 0.01 and *** *p* < 0.001 compared with the LPS group; ns is not significant.

The cytocompatibility of the CBD-ASD hydrogel was assessed using HDF cells exposed to hydrogel-conditioned media containing equivalent CBD concentrations. As illustrated in Figure 7a, the metabolic activity of the cells showed no substantial decline across the entire dosage range in comparison to the negative control. Notably, even at the maximum concentration tested, the survival rate of the cells persisted above 90%. This suggests that the prolonged delivery of amorphous CBD from the polymeric matrix does not elicit immediate toxicity, thereby confirming the excellent biocompatibility of the formulation. Moreover, hydrogel-based sustained delivery systems have been shown to mitigate burst-induced cytotoxicity by regulating drug exposure kinetics [6]. The results support the cytocompatibility of the CBD-ASD hydrogel and support its subsequent investigation into dermatological therapies.

### 3.11. Wound-Healing Activity of CBD-ASD Hydrogel

The CBD-ASD hydrogel’s ability to heal wounds was assessed in HDF cells using a scratch assay (Figure 7b,c). Following LPS-induced inflammatory stimulation, wound closure was monitored 18 h after treatment with conditioned media derived from hydrogels containing 5 µM and 10 µM CBD-ASD. Both formulations significantly enhanced cell migration compared to the hydrogel groups that were CBD-free and LPS-only, with 10 µM formulation exhibiting the most pronounced closure rate. Cannabidiol has been reported to promote keratinocyte and fibroblast migration through modulation of inflammatory signaling and oxidative stress pathways [51]. The enhanced regenerative response observed in this study suggests that incorporating CBD in an amorphous form enables sustained bioavailability, thereby facilitating cellular migration and wound repair.

### 3.12. Modulation of Inflammatory Gene Expression by CBD-ASD Hydrogel

**Figure 8:**
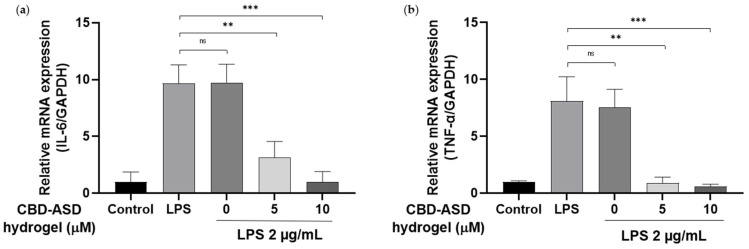
Quantitative RT-qPCR assessment of inflammatory gene transcription. Relative mRNA expressions of (**a**) IL-6 and (**b**) TNF-α treated with CBD-ASD hydrogel conditioned medium, excluding the untreated control group, cells were pretreated with LPS (2 µg/mL) for 2 h and subsequently cultured for 18 h in conditioned medium derived from CBD-ASD hydrogels containing 5 and 10 µM CBD. Results are expressed as mean ± standard deviation (*n* = 3). Statistical significance is indicated as ** *p* < 0.01 and *** *p* < 0.001 compared with the LPS group; ns is not significant.

The anti-inflammatory effects of the CBD-ASD hydrogel were examined via RT-qPCR analysis of pro-inflammatory cytokines such as IL-6 and TNF-α in HDF cells stimulated with LPS (Figure 8). LPS stimulation markedly increased cytokine expression compared with healthy controls. Treatment with conditioned media from CBD-ASD hydrogels (5 µM and 10 µM) significantly downregulated TNF-α and IL-6 expression compared to the CBD-free hydrogel and LPS-only groups. Notably, 10 µM formulation restored cytokine levels close to baseline values. CBD has been extensively demonstrated to attenuate the production of pro-inflammatory cytokines by regulating NF-κB signaling pathways and oxidative stress-associated responses [47,52]. The observed attenuation of gene expression indicates that sustained release of amorphous CBD from the hydrogel matrix effectively mitigates inflammatory activation in dermal fibroblasts. A CBD-ASD was successfully integrated into a borate-crosslinked PVA hydrogel to develop a structurally stable and multifunctional transdermal platform. Solid-state analyses confirmed effective amorphization of CBD, while rheological and swelling studies demonstrated a mechanically robust and well-regulated hydrogel network.

## 4. Conclusions

The ASD formulation markedly enhanced CBD release and skin permeation compared with crystalline CBD, reflecting improved solubility-driven diffusion and enhanced drug’s thermodynamic activity in the hydrogel matrix. The absence of crystalline lattice constraints facilitated more efficient drug partitioning and sustained transport across the skin-mimicking barrier. The hydrogel demonstrated concentration-dependent antioxidant and antibacterial activities, together with high cytocompatibility in dermal fibroblasts. The formulation promoted wound closure and significantly suppressed pro-inflammatory cytokine expression, indicating its capacity to modulate oxidative stress and inflammatory signaling while maintaining cellular viability. These combined physicochemical and biological effects underscore the multifunctional therapeutic potential of the system. The integration of amorphous solid dispersion technology with a dynamically crosslinked hydrogel matrix establishes a rational and adaptable therapeutic strategy to overcome the intrinsic solubility limits of water-insoluble cannabinoids. By enhancing bioavailability while enabling controlled and sustained delivery, this platform supports the development of advanced dermal and transdermal therapies for inflammatory skin disorders. Nevertheless, long-term physicochemical stability of the ASD formulation under varying storage conditions remains to be systematically evaluated in future studies. While the present study demonstrates the potential of the CBD-ASD hydrogel system through in vitro and ex vivo models, further in vivo investigations using relevant inflammatory skin disease models are warranted to fully evaluate its therapeutic efficacy and translational potential. These studies are planned as the next phase of our research.

## Figures and Tables

**Table 1:** Formulations of hydrogels. The hydrogels were prepared using polyvinyl alcohol (PVA), polyvinylpyrrolidone (PVP), boric acid (BA), chitosan (CS), CBD and distilled water (DW), as listed in the table. All values are presented in milligrams (mg) unless noted otherwise.

Samples	Solid Dispersion		PVA (mg)	CS (mg)	BA (mg)	DW (mL)
	CBD (mg)	PVP (mg)				
ASD hydrogel	0	750	750	20	53	6
CBD-ASD hydrogel BA-free	10	750	750	20	0	6
CBD-ASD hydrogel	10	750	750	20	53	6
CBD hydrogel	10	0	750	20	53	6

**Table 2:** Primer sequences used for RT-qPCR.

Target Genes	Primer Sequence
GAPDH	Forward: 5′-GAAGGTGAAGGTCGGAGTC-3′ Reverse: 5′-GAAGATGGTGATGGGATTTC-3′
IL-6	Forward: 5′-CCACGGCCTTCCCTACTTC-3′ Reverse: 5′-TTGGGAGTGGTATCCTCTGTGA-3′
TNF-α	Forward: 5′-AGGGTCTGGGCCATAGAACT-3′ Reverse: 5′-CCACCACGCTCTTCTGTCTAC-3′

**Table 3:** Kinetic parameters calculated from drug release data of CBD-ASD hydrogel and CBD hydrogel (*n* = 3).

Formulations	Zero-Order		First-Order		Higuchi		Hixson–Crowell		Korsmeyer–Peppas		
	*R* ^2^	*k*_0_	*R* ^2^	*k*_1_	*R* ^2^	*k*_H_	*R* ^2^	*k*_HC_	*R* ^2^	*n*	*k*_KP_
CBD-ASD hydrogel	0.720	0.473	0.900	−0.006	0.951	6.482	0.881	0.013	0.980	0.283	0.211
CBD hydrogel	0.904	0.170	0.924	−0.001	0.974	2.130	0.961	0.003	0.920	0.302	0.060

**Table 4:** Antibacterial activity of the CBD-ASD hydrogels. Comparison of inhibition zone diameters of the three hydrogel treatments at different concentrations against *S. aureus* bacteria. Data are presented as mean ± SD (*n* = 3).

Samples	ASD Hydrogel	CBD-ASD Hydrogel		
		L (10 mg)	M (75 mg)	H (100 mg)
Diameter (cm)	1.86 ± 0.27	2.13 ± 0.16	2.30 ± 0.24	3.93 ± 0.93

## Data Availability

All relevant data are contained within the article. Requests for additional information should be addressed to the corresponding author.

## Abbreviations

The following abbreviations are used in this manuscript:

AD	Atopic dermatitis
ASD	Amorphous solid dispersion
CBD	Cannabidiol
CBD-ASD	Cannabidiol-loaded amorphous solid dispersion
CFU	Colony-forming unit
CS	Chitosan
DSC	Differential scanning calorimetry
DPPH	2,2-Diphenyl-1-picrylhydrazyl
FE-SEM	Field emission scanning electron microscopy
FT-IR	Fourier-transform infrared spectroscopy
GAPDH	Glyceraldehyde-3-phosphate dehydrogenase
HDF	Human dermal fibroblast
HPLC	High-performance liquid chromatography
IL-6	Interleukin-6
LPS	Lipopolysaccharide
PVA	Polyvinyl alcohol
PVP	Polyvinylpyrrolidone
PXRD	Powder X-ray diffraction
RT-qPCR	Reverse transcription quantitative polymerase chain reaction
SEM	Scanning electron microscopy
TNF-α	Tumor necrosis factor-alpha
